# Health education for microcredit clients in Peru: a randomized controlled trial

**DOI:** 10.1186/1471-2458-11-51

**Published:** 2011-01-24

**Authors:** Rita Hamad, Lia CH Fernald, Dean S Karlan

**Affiliations:** 1 Joint Medical Program, University of California Berkeley - University of California San Francisco, Berkeley, USA; 2 School of Public Health, University of California Berkeley, Berkeley, USA; 3 Department of Economics, Yale University, New Haven, USA

## Abstract

**Background:**

Poverty, lack of female empowerment, and lack of education are major risk factors for childhood illness worldwide. Microcredit programs, by offering small loans to poor individuals, attempt to address the first two of these risk factors, poverty and gender disparity. They provide clients, usually women, with a means to invest in their businesses and support their families. This study investigates the health effects of also addressing the remaining risk factor, lack of knowledge about important health issues, through randomization of members of a microcredit organization to receive a health education module based on the World Health Organization's Integrated Management of Childhood Illness (IMCI) community intervention.

**Methods:**

Baseline data were collected in February 2007 from clients of a microcredit organization in Pucallpa, Peru (n = 1,855) and their children (n = 598). Loan groups, consisting of 15 to 20 clients, were then randomly assigned to receive a health education intervention involving eight monthly 30-minute sessions given by the organization's loan officers at monthly loan group meetings. In February 2008, follow-up data were collected, and included assessments of sociodemographic information, knowledge of child health issues, and child health status (including child height, weight, and blood hemoglobin levels). To explore the effects of treatment (i.e., participation in the health education sessions) on the key outcome variables, multivariate regressions were implemented using ordinary least squares.

**Results:**

Individuals in the IMCI treatment arm demonstrated more knowledge about a variety of issues related to child health, but there were no changes in anthropometric measures or reported child health status.

**Conclusions:**

Microcredit clients randomized to an IMCI educational intervention showed greater knowledge about child health, but no differences in child health outcomes compared to controls. These results imply that the intervention did not have sufficient intensity to change behavior, or that microcredit organizations may not be an appropriate setting for the administration of child health educational interventions of this type.

**Trial Registration:**

This study is registered with ClinicalTrials.gov, NCT01047033.

## Background

### A Comprehensive Intervention to Reduce Childhood Illness

Since 1995, the Division of Child Health and Development at the World Health Organization has been partnering with governments and non-governmental organizations to promote a comprehensive strategy to address the multi-factorial and interactive determinants of childhood illness [1]. Known as the Integrated Management of Childhood Illness (IMCI), this program's goal is to reduce death, illness, and disability among children less than five years old, while supporting their growth and development [2]. The three goals of IMCI are to improve a country's infrastructure, to train health workers, and to deliver community-based interventions such as health education. The issues addressed by the three components of IMCI are modified based on the needs of the region of delivery, but generally include breastfeeding promotion, and the prevention and treatment of pneumonia, diarrhea, malaria, measles, and malnutrition through various techniques [1].

Studies in many developing countries have shown IMCI to be successful at improving child health outcomes. Some have assessed interventions encompassing all three IMCI components, while others have assessed only one or two. A large cluster-randomized controlled longitudinal trial in rural Bangladesh evaluated a five-year intervention involving all three components of IMCI, and included training of health providers, household visits by trained health workers, and mosque-based sermons on IMCI-related topics. The study found that communities receiving IMCI had significantly lower rates of stunting (57% vs. 50%) and higher rates of exclusive breastfeeding (76% vs. 65%) than those in the treatment group, in part due to improvements in healthcare provision [3]. An observational study of communities in Tanzania receiving the health worker training component of IMCI found more appropriate diagnosis and treatment of children in health centers compared to control communities after a treatment period of three years, as well as increases in health knowledge among parents [4]. No randomized trials have separated the independent effects of the community-based educational component of IMCI.

### Integration of Microcredit and Health Education

An early multi-country evaluation of IMCI in Bangladesh, Brazil, Peru, Tanzania, and Uganda concluded that the community component of IMCI should include not just government health facilities but a broader range of channels in order to increase the potential reach of delivery systems [5]. Since then, a diverse group of non-governmental organizations (NGOs), including several microcredit organizations, has begun to incorporate IMCI-like community interventions into their strategies for improving child health, either as official partners with national IMCI programs or on their own [6,7].

Microcredit is an economic intervention involving the provision of small loans to clients - typically women - who are too poor to borrow from traditional lending institutions. Microcredit institutions are diverse, run by governments, not-for-profit groups, or banks, and operate throughout the developing world [8]. Since microcredit's inception, there has been great interest in the potential of these small loans to improve the health and wellbeing of a client's family and children [9]. Loans are often given to groups of clients rather than to individual borrowers, with the assumption that group members will provide one another with social collateral in place of physical collateral. Some microcredit organizations also offer supplemental services to borrowers, such as education or healthcare, promoting both the social and economic development of clients [10]. With their wide distribution and access to poor individuals throughout the world, microcredit institutions are well positioned to implement educational interventions such as IMCI [11]. Combining IMCI with the economic benefits of microcredit may lead to greater improvements in child health and development outcomes, given that poverty is a major underlying cause of disease in the developing world.

Several authors have examined the impact of microcredit on child health outcomes, although there is little similarity in the survey or analysis techniques used across studies. One study in the Dominican Republic compared three villages - one in which a microcredit program operated, one in which a health promotion program operated, and another in which both programs operated - and found that higher rates of childhood vaccination could be attributed to the presence of a microcredit program, although there was no difference in rates of diarrhea or acute respiratory infection [12]. Studies in Ghana, Bolivia, and Bangladesh that compared clients receiving microcredit and health education to comparison groups that received neither intervention found improvements among the treatment group in a variety of indicators, such as health knowledge among parents, feeding frequency and rehydration following a child's diarrheal episode, and child height-for-age [13-15]. These studies, however, did not distinguish between the differential effects of the microcredit and educational components of the intervention.

One observational study conducted in Ecuador and Honduras compared clients receiving only microcredit to those receiving microcredit plus health education. Clients who received an additional health education component in Honduras had a statistically significant decrease in childhood diarrhea incidence, whereas there was no statistically significant difference among banking-only clients. In Ecuador, banking-only clients experienced a reduction in diarrhea incidence with no statistically significant difference in incidence among clients who received an additional health education component [16].

### Aims and Hypotheses

In this study we used a randomized design to investigate the effects of an IMCI-based health education intervention on child health outcomes and parental knowledge among clients of PRISMA, a microcredit organization in Pucallpa, Peru. We hypothesized that participation in health education sessions would improve clients' knowledge of child health issues, and that this involvement would translate into healthier development and decreased illness among children of clients. This study is novel in investigating the effect of IMCI in the setting of an economic intervention, in which the potentially increased income and empowerment provided by the microcredit may increase clients' ability to act on the information gleaned from the educational sessions. Our sample was diverse with respect to sociodemographic variables, which allows us to investigate whether outcomes differ based on client characteristics. As female clients have previously been shown to invest more resources in their children than male clients [17], we hypothesized that positive effects would be larger among women and their children. The children of younger, less educated, or less wealthy parents may also benefit more from the educational sessions [18].

## Methods

### Country Context of This Study

This study was conducted in Peru, one of several countries in Latin America undergoing demographic and epidemiological transitions. Despite improvements in the nation's healthcare system and infrastructure and a shift towards non-communicable diseases, infectious disease remains the leading cause of death [19]. In 2001, 43% of total mortality among children aged 0-4 years was due to communicable diseases such as respiratory and intestinal infections [20]. A study conducted by Peru's National Institute of Statistics and Information Technology (INEI) in conjunction with the Demographic and Health Surveys in 2004 found that 15.1% of children in the preceding two weeks were reported to have diarrhea, and 17.3% had had respiratory infections [21].

### Study Design and Sampling

This study was carried out from February 2007 to February 2008 in collaboration with PRISMA, an NGO that provides microcredit loans to clients throughout Peru, in addition to other services such as agricultural development and health education. Our research was conducted in and around the city of Pucallpa, a city in the jungle region of eastern Peru. Pucallpa has a population of about 136,000, with 93% of residents classified as urban and 7% as rural [22].

The Pucallpa branch of PRISMA had a total of 2,134 clients at baseline. Clients organize themselves into loan groups of 10 to 20 members each. While some groups consisted entirely of women, others included both men and women. Groups met with a loan officer once a month at PRISMA's offices or at the home of a client to re-pay their loans and participate in other activities as needed. There were 139 loan groups at baseline, each overseen and administered by one of four loan officers hired by PRISMA. At baseline, this branch provided no services beyond the lending services.

Innovations for Poverty Action, a US-based non-profit that worked with PRISMA to conduct this study, hired and trained local Spanish-speaking surveyors. A questionnaire was developed that included questions on a variety of indicators, described below, and was translated into Peruvian Spanish by native speakers fluent in both English and Spanish. Focus groups took place among a small group of clients to ensure that questions were understandable, and validity testing was conducted.

**Figure 1:**
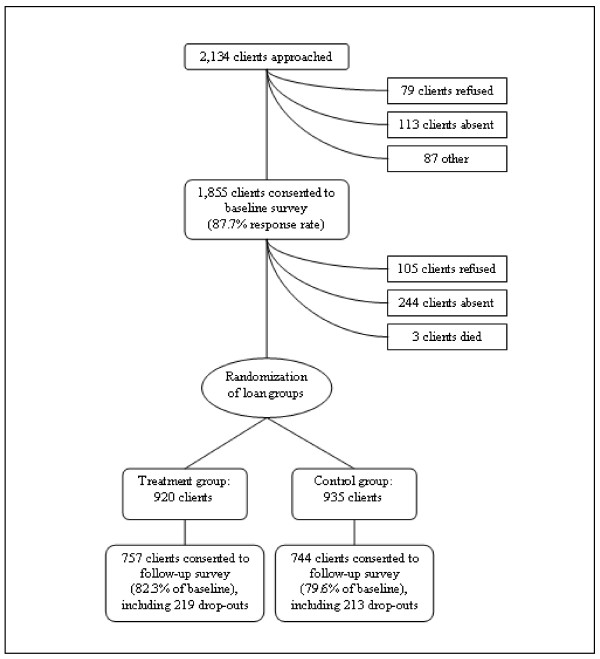
**Sampling framework**.

In February 2007, surveyors approached all clients in Pucallpa after their monthly group meetings to conduct interviews individually or to schedule an interview at the client's home. Surveyors telephoned clients who were not present in order to schedule an appointment. The questionnaire, described in detail below, was then administered. Of the 2,134 clients served by the Pucallpa branch, 1,855 consented to complete the survey, for a response rate of 87.7% (Figure 1). The primary reasons for non-response included client absence (113 clients) or refusal (79 clients). Anthropometric measurements and information about health status were obtained for 598 children under the age of five years.

### Process of Randomization

After the baseline survey was completed, half of loan groups were randomized to receive an additional health education intervention, described below, in addition to the existing microcredit services. The remaining loan groups continued to receive only microcredit services. Randomization of loan groups was conducted using a computerized random number generator and implemented by research assistants based in Peru.

In January and February 2008, surveyors once again approached clients. Of the 1,855 clients from the baseline survey, 1,501 (81.0%) consented to participate (Figure 1). The remainder refused to complete the survey (105 clients), were unable to be located (244 clients), or had died (3 clients). The NGO's records indicate that at least 156 (44.3%) of those clients who refused or could not be located had dropped out of their loan groups at some point between the baseline and follow-up surveys. On the other hand, 432 clients who had dropped out of their loan groups did complete the follow-up survey, and among these clients, there was no significant difference in loan group drop-out rates between those in the treatment (29.3%) and control (29.4%) groups. Clients provided information for 454 (75.9%) of the children that had been evaluated at baseline.

In accordance with the recommendations of the CONSORT Group [23], an initiative to promote transparent reporting of parallel-group randomized controlled trials, we have completed the standard CONSORT checklist and flow diagram to provide further details of the implementation of this study and the interpretation of its results (Additional Files 1 and 2).

### Ethics Approval

Ethics approval was obtained from the Committee for the Protection of Human Subjects at the University of California Berkeley and the PRISMA and Innovations for Poverty Action ethics committees. Participants gave their written consent to take part in the study. Those with children under the age of five years gave consent on behalf of their children, and children were also asked for their verbal assent before being included in the study. This trial is registered with ClinicalTrials.gov (NCT01047033).

### Description of the Intervention

PRISMA provides microcredit services to urban and rural individuals and households throughout Peru. It charges a 4% monthly interest, calculated on the original balance of the loan, in addition to a 1% commission when the loan is disbursed. The loan size depends primarily on how long the client has been a member of the NGO, with the size increasing incrementally with each loan cycle. The mean loan size among clients in Pucallpa is US$451 (range: US$106 to US$2468) repaid over a period of six months. At the end of six months, most clients continue their relationship with PRISMA and their loan group to receive additional loans, although the organization does not keep data to calculate the percentage of clients who drop out.

With the goal of creating a positive social impact on clients, PRISMA often provides services to microcredit clients and other community members at its branches, ranging from support for agricultural development to education modules on a variety of subjects. The organization agreed to randomize the IMCI child health education module to loan groups in Pucallpa as part of its commitment to evidence-based practice.

The intervention involved 30 minutes of health education, facilitated by loan officers at the end of monthly group meetings over the course of eight months. As each loan cycle is six months, the educational sessions spanned more than one loan cycle, during which clients remained with their loan group to receive an additional loan. PRISMA's US-based affiliate, Freedom From Hunger, developed these sessions based on the community component of the IMCI strategy developed by the World Health Organization [2]. During each monthly meeting, loan officers presented basic information on child health to clients and provided an opportunity for the men and women to discuss their own experiences and identify appropriate solutions. Examples of topics covered included diarrhea, cough, and fever, as well as information about interactions with healthcare providers in order to empower caregivers during clinic visits.

### Measures

#### Treatment Variable

The treatment group was represented by a binary variable corresponding to whether the client's loan group was randomized to receive health education.

#### Sociodemographic Variables

Clients were asked about their age, gender, and educational attainment at the time of the baseline study. We hypothesized that the effects of education and age would be non-linear, and that an attainment of a minimum threshold of age and educational status would be more important as a predictor variable than incremental effects would be. Therefore age and educational status were split at the 25^th ^percentile to create binary variables representing clients who were younger than 32 years and clients who had not yet completed primary education, respectively. Clients also provided the age (in months) and gender for each of their children below five years of age at the time of the baseline study. Information was obtained on assets owned by the household, such as televisions, cars, and refrigerators, using questions adapted from Demographic and Health Surveys [24]. As in previous studies, principal component analysis (PCA) was used to construct one variable representing household assets as a proxy for relative poverty among households [25,26]. As above, this variable was then split at the 25^th ^percentile to create a binary variable representing clients with fewer household assets.

#### Loan Officer Quality

The results of a separate qualitative evaluation, not shown here, suggested that some of the four loan officers were more skilled at facilitating the health education sessions than others, demonstrating more knowledge about health topics and a greater ability to engage the clients during the sessions. Based on these findings, a binary variable was created to indicate whether a client's loan officer was skilled or unskilled.

#### Outcome Variables

##### Health Knowledge

Clients were asked a series of questions to assess their knowledge about childhood illness. For example, they were asked to identify threatening situations that might lead them to take their child to a clinic, such as if the child had bloody diarrhea, vomiting, or difficulty breathing. They were also asked to identify appropriate treatments for these conditions, such as giving a child increased fluids for diarrhea. These data were only collected at follow-up, and the questions were based on prior surveys conducted by Freedom From Hunger.

##### Child Health Status

Clients were asked a series of questions about the health of each of their children under the age of five years, based on the content of the intervention. For example, they were asked how many days of diarrhea the child had experienced in the last four weeks, or whether they had had difficulty breathing due to a cough. These data were only collected at follow-up. Additionally, height and weight were collected at baseline and follow-up using standard techniques [27]. Wooden stadiometers were constructed locally to measure height, and digital Taylor Electronic Lithium Scales Model 7324W recorded weight. Z-scores for each child's weight-for-age, length/height-for-age, and body mass index (BMI)-for-age were calculated using WHO reference standards [28]. These were then used to determine the prevalence of underweight, stunting, and overweight in this population. Blood hemoglobin levels were measured at baseline and follow-up with finger pricks using the HemoCue Hb 201+ System (HemoCue Inc., Lake Forest, CA). Hemoglobin levels were not adjusted for altitude because Pucallpa lies at 200 m above sea level [29].

### Data Analysis

Data were double-entered using CSPro 3.3 (U.S. Census Bureau, Population Division, Washington, D.C.). Statistical analyses were conducted using STATA SE 10.1 for Windows (STATA Corporation, College Station, TX).

Balance between the treatment and control groups was tested using t-tests or tests of proportions, as appropriate. To explore the effects of treatment (i.e., participation in the health education sessions) on the key outcome variables, multivariate regressions were implemented using ordinary least squares. The predictor variable was a binary variable corresponding to randomization into the treatment or control arm.

Each regression controlled for the sociodemographic variables listed above. Where possible, we also controlled for the baseline value of the outcome variable. Standard errors were calculated allowing for clustering within the loan group, the unit of randomization. Similarly, regressions of child-related outcomes allowed for standard errors to be clustered at the level of the parent, as several clients had more than one child that participated in the survey. Interactions between treatment group and client age, gender, education, and household assets were conducted using standard techniques [30]. We also included an interaction between the treatment group and the skill level of the loan officer.

## Results

### Characteristics of Clients and Their Children

Clients randomized to the two study arms were balanced with respect to most demographic and socioeconomic characteristics at baseline (Table 1). Children of clients in the treatment and control groups were balanced with respect to age, gender, and anthropometric measures (Table 2).

**Table 1:** Client characteristics at baseline

Variable	Study Arm		Test of proportions or t-test
	
Demographic Characteristics	Control (n = 935)	Treatment (n = 920)	
Gender (%)			
Male	14.9	13.2	0.30
Female	85.1	86.8	

Age (%)			
< 30 years	17.1	18.4	0.47
30-39 years	34.8	34.4	0.87
40-49 years	30.1	29.3	0.71
50-59 years	15.3	15.3	0.97
≥60 years	0.03	0.03	0.93

No. of children < 5 yrs old surveyed (mean ± SD)	0.35 ± 0.59	0.31 ± 0.53	0.12

**Socioeconomic Characteristics**			

Education (%)			
Primary or less	26.9	26.1	0.69
Incomplete secondary	24.7	28.4	0.08
Complete secondary	26.4	25.8	0.77
Post-secondary	21.9	19.7	0.24

Household assets, PC ^a ^(mean ± SD)	-0.12 ± 1.6	0.07 ± 1.8	0.02

^a ^Principal component

**Table 2:** Child characteristics at baseline

Variable	Study Arm		Test of proportions or t-test
	
Demographic Characteristics	Control (n = 319)	Treatment (n = 279)	
Gender (%)			
Male	47.4	52.3	0.24
Female	52.6	47.7	

Age (in years; mean ± SD)	2.6 ± 1.4	2.6 ± 1.4	0.66

**Child Anthropometric Measures **^a^			

Hemoglobin, g/dL	10.9 ± 1.2	10.9 ± 1.3	0.91

Weight-for-age, Z-score	-0.60 ± 1.4	-0.73 ± 1.3	0.26

Length/height-for-age, Z-score	-1.2 ± 1.4	-1.4 ± 1.4	0.09

BMI-for-age, Z-score	0.31 ± 1.4	0.24 ± 1.3	0.61

^a ^Results are given as mean ± SD

### Analysis of Effect of IMCI on Child Health

At the one-year follow-up, clients whose loan groups were randomized to receive the education sessions were significantly more knowledgeable on a variety of issues related to child health (Table 3). Analyses of interaction terms revealed that less educated parents who were randomized to the treatment group demonstrated more knowledge about doctor's office activities than more educated parents who received treatment. There were no other significant heterogeneous treatment effects with respect to client age, gender, or education, or child age and gender (data not shown), although sample size on subsamples would only detect large differences.

**Table 3:** Caregiver health knowledge at follow-up, by study arm

	Control	Treatment	β-coefficient (95% CI)	n	p-value ^a^
**Caregiver Health Knowledge:** **Percent of clients able to report at least one item in each category **					

Diarrhea danger signs	85.3	90.0	0.12 (0.07, 0.18)	1447	< 0.01

Dietary components for child w/diarrhea	74.3	82.7	0.11 (0.04, 0.17)	1383	< 0.01

Elements of changes in care for child w/diarrhea	89.6	93.3	0.10 (0.03, 0.18)	1472	0.06

Cough danger signs	96.8	96.3	0.05 (-0.01, 0.10)	1467	0.64

Other danger signs	76.2	82.7	0.09 (0.00, 0.17)	1312	0.02

Knowledge of doctor's office activities	92.9	96.6	0.18 (0.05, 0.31)	1435	< 0.01

Knowledge of important activities after doctor's appointment	97.2	98.4	0.04 (-0.02, 0.11)	1477	0.07

^a ^Multivariate analyses clustered at the group level and controlling for client gender, age, and education, household assets, and loan officer.

Child anthropometric measures and reported health status were not significantly different between the two groups (Table 4). Analyses of interaction terms revealed that the children of clients who received treatment from a skilled loan officer had reduced bloody diarrhea in comparison to the children of those who had an unskilled loan officer. There were no significant heterogeneous treatment effects with respect to client age, gender, or education, or child age and gender (data not shown), although sample size on subsamples would only detect large differences.

**Table 4:** Child anthropometric measures and health status at follow-up, by study arm

	Control	Treatment	β-coefficient (95% CI)	n	p-value ^a^
**Child Anthropometric Measures **^**b**^					

Hemoglobin, g/dL	11.5 ± 1.2	11.5 ± 1.1	0.01 (-0.23, 0.26)	312	0.94
Anemia ^c ^(%)	49.2	48.0	0.02 (-0.08, 0.12)	312	0.70

Weight-for-age, Z-score	-0.71 ± 1.1	-0.73 ± 1.0	-0.02 (-0.26, 0.23)	309	0.87
Underweight ^d ^(%)	10.6	7.7	-0.04 (-0.11, 0.03)	301	0.28

Length/height-for-age, Z-score	-1.3 ± 1.3	-1.4 ± 1.6	-0.14 (-0.51, 0.23)	283	0.46
Stunting ^e ^(%)	24.3	28.4	0.01 (-0.08, 0.11)	264	0.77

BMI-for-age, Z-score	0.13 ± 1.3	0.29 ± 1.4	0.18 (-0.15, 0.51)	290	0.29
Overweight ^f ^(%)	17.5	25.7	0.05 (-0.05, 0.15)	265	0.31

**Reported Child Health Status ^g^**					

Diarrhea	15.8	22.3	0.05 (-0.03, 0.13)	439	0.19

Bloody diarrhea	15.3	4.2	-0.11 (-0.24, 0.02)	85	0.09

Days of diarrhea (mean ± SD)	3.6 ± 5.0	2.6 ± 1.6	-1.04 (-2.54, 0.45)	99	0.17

Diarrhea requiring medical attention	38.4	40.8	0.02 (-0.21, 0.25)	86	0.88

Cough in past month	36.7	40.2	0.02 (-0.07, 0.12)	434	0.61

Days of cough (mean ± SD)	5.4 ± 3.6	5.3 ± 3.6	-0.01 (-1.14, 1.13)	175	0.99

Cough causing difficulty breathing	33.7	33.7	-0.01 (-0.16, 0.14)	167	0.90

Cough requiring medical attention	58.4	58.1	0.05 (-0.11, 0.21)	166	0.57

^a ^Multivariate analyses clustered at the parent level controlling for child age and gender, client gender, age, and education, household assets, and loan officer.

^b ^Results are given as mean ± SD, unless noted otherwise. These analyses also controlled for the baseline value of the variable in question.

^c ^A hemoglobin of < 11 g/dL is considered anemic [29].

^d ^Underweight is a weight-for-age > 2 SD below the WHO reference [28].

^e ^Stunting is a length-for-age > 2 SD below the WHO reference [28].

^f ^Overweight is a BMI above the 85^th ^percentile for the child's age [31].

^g ^Results are given as prevalence during past month (%), unless noted otherwise.

## Discussion

This results of this study indicate that randomization to a health education intervention based on the WHO's IMCI strategy improved health knowledge in a large sample of microcredit clients in urban Peru on some issues related to child health. There were no differences in child anthropometric measures or reported child health status. Less educated parents demonstrated more knowledge than more educated parents on one aspect of child health, and children of clients who received treatment from skilled loan officers were less likely to have bloody diarrhea than those whose loan officers were unskilled.

Previous evaluations of IMCI have involved assessments of all three program components combined [3], or assessments of just the health worker training component or the community education components in isolation [32-34]. Of those examining the community education component, one study in Armenia showed improvements in both parental knowledge and practice [35], while studies in Peru, Honduras, and Ethiopia demonstrated improvements in child health outcomes as well [36,37].

We propose several explanations why this evaluation did not alter child health status despite improvements in parental knowledge. Traditional models of health promotion suggest that, once individuals gain increased knowledge about a health concept, they must then alter their attitudes and behaviors before a change is seen in the outcome [38]. Our study only measured changes in client knowledge and health outcomes, and may have failed to capture whether attitudes and behaviors among study subjects were impacted. It is also possible that one component of IMCI considered in isolation was not sufficient to change behaviors and outcomes in this population, as the local healthcare infrastructure remained unchanged and possibly inadequate. This suggestion was also made by a previous study in Brazil that found no changes in child health indicators after implementation of an isolated IMCI health worker training intervention [32].

While a previous study of a community IMCI program in Peru found that child health outcomes improved in the absence of the other IMCI components [37], the context of the intervention and the delivery methods differed significantly from those of this study. The previous study took place among a community in Peru in which the majority of the residents are employed in the agricultural industry, a population that is less educated and less financially stable than our sample of urban-based microentrepreneurs [22]. That evaluation also involved interactive home visits to individual families by local Red Cross personnel rather than monthly group education sessions by microcredit loan officers. The benefits of taking advantage of the existing organizational structure that a group of microcredit clients provides may be outweighed by a loss of individualized attention to each parent's needs and a decrease in the intensity of the intervention.

Also, while loan officers in this study were trained to carry out the education sessions, they did not have the extensive health training that a member of the Red Cross possesses. In informal interviews with the loan officers at the Pucallpa branch, all expressed frustration at being requested to educate clients about information with which they were not very familiar, and without financial compensation for spending the extra time to do so. In observing several client meetings, the authors of this study noticed a variation in the quality of the educational sessions being conducted by the various loan officers. This finding was confirmed in secondary analyses (not shown), in which clients of particular loan officers consistently ranked significantly lower in knowledge than those of other loan officers. As described above, we also found that parents whose loan officers were skilled reported less bloody diarrhea in their children than those whose loan officers were less skilled.

Another large study of IMCI in Peru found that child health indicators did not improve [39]. They noted that, in contrast to Bangladesh and Tanzania, where prior studies of IMCI have been successful [3,4], Peru has lower infant mortality rates and a population with a relatively higher socioeconomic status. Moreover, clients in this study demonstrated a higher socioeconomic status than the average Peruvian population (data not shown), with 93% of clients reporting electricity in their homes compared to 73% nationally, and higher average educational attainment compared to a national sample [21]. Consequently, these individuals may not benefit as much from an intervention targeted primarily to poorer populations [39]. While our study found increased client knowledge among those in the treatment group, overall awareness of child health issues was already high. Lack of knowledge therefore may not be the limiting factor for child morbidity, or a more sophisticated intervention may be more appropriate in this setting.

## Conclusions

Our study suggests that a child health educational intervention based on the WHO's IMCI strategy was not an effective method to improve child health outcomes among microcredit clients in a major urban center in Peru. Various aspects of the IMCI strategy have been found to be successful in other settings and should be pursued; this study, however, joins a few others in demonstrating that some variations of this intervention, particularly if they are not intensive, may not be effective. Moreover, while microcredit is being proposed worldwide as a powerful economic intervention and as a strong method through which to deliver other supplemental programs, there are few randomized controlled trials to support these claims and more studies are needed to rigorously evaluate whether microcredit offers a promising platform for delivering supplemental services.

## Competing interests

The authors declare that they have no competing interests.

## Authors' contributions

RH designed the study, traveled to Peru to supervise data collection, analyzed and interpreted the data, conducted the literature search, and was the primary author of the manuscript. LCHF designed the study, interpreted the data, contributed towards the literature search, and helped author the manuscript. DSK designed the study, traveled to Peru to supervise data collection, interpreted the data, and helped author the manuscript. All authors read and approved the final manuscript.

## Funding

The American Women's Hospitals Services, the Bixby Program at the University of California Berkeley (UCB), the Center for Latin American Studies at UCB, the Dean's Summer Fellowship at the University of California San Francisco (UCSF), the Human Rights Center at UCB, the Interdisciplinary MPH Program at the UCB School of Public Health, the Rainer Fund, the UCSF-UCB Joint Medical Program, and the Bill and Melinda Gates Foundation. Study funders had no role in the study design; in collecting, analyzing, or interpreting the data; in writing the report; or in the decision to submit the article for publication.

## Pre-publication history

The pre-publication history for this paper can be accessed here:

http://www.biomedcentral.com/1471-2458/11/51/prepub

## Supplementary Material

Additional file 1 **CONSORT Flow Diagram**. As described in the Methods section, we have included the standard CONSORT checklist and flow diagram to provide further details of the implementation of this study and the interpretation of its results. Click here for file

Additional file 2 **CONSORT Checklist**. As described in the Methods section, we have included the standard CONSORT checklist and flow diagram to provide further details of the implementation of this study and the interpretation of its results. Click here for file

## References

[B1] WHO Improving child health. IMCI: the integrated approach 1997 Washington, DC: World Health Organization

[B2] Integrated Management of Childhood Illness http://www.who.int/child_adolescent_health/topics/prevention_care/child/imci/en/index.html

[B3] Arifeen S Hoque D Akter T Rahman M Hoque M Begum K Chowdhury E Khan R Blum L Ahmed S Effect of the Integrated Management of Childhood Illness strategy on childhood mortality and nutrition in a rural area in Bangladesh: a cluster randomised trial Lancet 2009 374 9687 393 403 10.1016/S0140-6736(09)60828-X 19647607

[B4] Armstrong Schellenberg J Bryce J de Savigny D Lambrechts T Mbuya C Mgalula L Wilczynska K The effect of Integrated Management of Childhood Illness on observed quality of care of under-fives in rural Tanzania Health Policy Plan 2004 19 1 1 10 10.1093/heapol/czh001 14679280

[B5] Bryce J Victora CG Habicht JP Black RE Scherpbier RW Programmatic pathways to child survival: results of a multi-country evaluation of Integrated Management of Childhood Illness Health Policy Plan 2005 20 Suppl 1 i5 i17 10.1093/heapol/czi055 16306070

[B6] Child Survival Technical Support Project Reaching Communities for Child Health and Nutrition: A Framework for Household and Community IMCI 2001 Calverton, Maryland

[B7] Levinger B McLeod J A Wealth of Opportunity: Partnering with CORE and CORE Group Members 2002 Washington, DC: CORE Group

[B8] Karlan DS Morduch J Rodrick D, Rosenzweig MR Access to Finance Handbook of Development Economics, 5 2010 Amsterdan

[B9] Yunus M Anderson CL, Looney JW Toward eliminating poverty from the world: Grameen Bank experience Making Progress: Essays in Progress and Public Policy 2002 Lanham, MD: Lexington Books 371 378

[B10] Simanowitz A Walter A Daley-Harris S Ensuring impact: reaching the poorest while building financially self-sufficient institutions, and showing improvement in the lives of the poorest women and their families Pathways Out of Poverty: Innovations in Microfinance for the Poorest Families 2002 Bloomfield, CT: Kumarian Press, Inc

[B11] Dunford C Daley-Harris S Building better lives: sustainable integration of microfinance with education in child survival, reproductive health, and HIV/AIDS prevention for the poorest entrepreneurs Pathways Out of Poverty: Innovations in Microfinance for the Poorest Families 2002 Bloomfield, CT: Kumarian Press, Inc

[B12] Dohn AL Chávez A Dohn MN Saturria L Pimentel C Changes in health indicators related to health promotion and microcredit programs in the Dominican Republic Revista Panamericana de Salud Pública 2004 15 3 185 193 10.1590/s1020-49892004000300007 15096291

[B13] Hadi A Promoting health knowledge through micro-credit programmes: experience of BRAC in Bangladesh Health Promotion International 2001 16 3 219 227 10.1093/heapro/16.3.219 11509457

[B14] MkNelly B Dunford C Impact of *Credit with Education *on Mothers and Their Young Children's Nutrition: Lower Pra Rural Bank *Credit with Education *Program in Ghana 1998 Davis, CA: Freedom From Hunger

[B15] MkNelly B Dunford C Impact of *Credit with Education *on Mothers and Their Young Children's Nutrition: CRECER *Credit with Education *Program in Bolivia 1999 Davis, CA: Freedom From Hunger

[B16] Smith SC Village banking and maternal and child health: evidence from Ecuador and Honduras World Development 2002 30 4 707 723 10.1016/S0305-750X(01)00128-0

[B17] Thomas D Intra-household resource allocation: an inferential approach The Journal of Human Resources 1990 25 4 635 664 10.2307/145670

[B18] Fernald LC Gertler P Neufeld L 10-year eff ect of Oportunidades, Mexico's conditional cash transfer programme, on child growth, cognition, language, and behaviour: a longitudinal follow-up study Lancet 2009 374 9706 1997 2005 10.1016/S0140-6736(09)61676-7 19892392

[B19] PAHO Health situation in the Americas: Basic indicators. Washington, D.C 2009

[B20] Mathers CD Lopez AD Murray CJL The Burden of Disease and Mortality by Condition: Data, Methods, and Results for 2001 Global Burden of Disease and Risk Factors 2006 New York: Oxford University Press 45 93 21250373

[B21] INEI/DHS Encuesta Demográfica y de Salud Familiar 2004. Lima 2005

[B22] INEI/DHS Censos Nacionales 2007: XI de Población y VI de Vivienda Lima 2007

[B23] Schulz K Altman D Moher D CONSORT Group CONSORT 2010 Statement: updated guidelines for reporting parallel group randomised trials BMC Medicine 2010 8 18 10.1186/1741-7015-8-18 PMC2860339 20334633

[B24] Demographic and Health Surveys http://www.measuredhs.com

[B25] Filmer D Pritchett L Estimating Wealth Effects without Expenditure Data--or Tears: An Application to Educational Enrollments in States of India. Policy Research Working Papers No. 1994 1998 Washington, DC: World Bank 10.1353/dem.2001.0003 11227840

[B26] Zeller M Houssou N Alcaraz GV Schwarze S Johannsen J Developing Poverty Assessment Tools based on Principal Component Analysis: Results from Bangladesh, Kazakhstan, Uganda, and Peru International Association of Agricultural Economists 2006 Annual Meeting. Queensland, Australia 2006

[B27] Habicht JP Estandarización de métodos epidemiológicos cuantitativos sobre el terreno [Standardization of quantitative epidemiological methods in the field] [Article in Spanish] Bol Oficina Sanit Panam 1974 76 5 375 384 4277063

[B28] WHO Physical status: the use and interpretation of anthropometry. Report of a WHO expert committee. Technical report series no. 854 Geneva 1995 854 8594834

[B29] WHO Iron Deficiency Anaemia: Assessment, Prevention, and Control. A Guide for Programme Managers Geneva 2001

[B30] Aiken L West S Multiple regression: Testing and interpreting interactions 1991 London: Sage Publications

[B31] Mei Z Grummer-Strawn LM Pietrobelli A Goulding A Goran M Dietz W Validity of body mass index compared with other body-composition screening indexes for the assessment of body fatness in children and adolescents Am J Clin Nutr 2002 75 6 978 985 12036802 10.1093/ajcn/75.6.978

[B32] Amaral J Leite AJ Cunha AJ Victora CG Impact of IMCI health worker training on routinely collected child health indicators in Northeast Brazil Health Policy Plan 2005 20 Suppl 1 i42 i48 10.1093/heapol/czi058 16306068

[B33] Rowe AK Onikpo F Lama M Osterholt DM Rowe SY Deming MS A multifaceted intervention to improve health worker adherence to integrated management of childhood illness guidelines in Benin Am J Public Health 2009 99 5 837 846 10.2105/AJPH.2008.134411 19299681 PMC2667861

[B34] Santos I Victora CG Martines J Goncalves H Gigante DP Valle NJ Pelto G Nutrition counseling increases weight gain among Brazilian children J Nutr 2001 131 11 2866 2873 11694610 10.1093/jn/131.11.2866

[B35] Thompson M Harutyunyan TL Impact of a community-based integrated management of childhood illnesses (IMCI) programme in Gegharkunik, Armenia Health Policy and Planning 2009 24 2 101 107 10.1093/heapol/czn048 19147699

[B36] Ali M Asefaw T Byass P Beyene H Pedersen FK Helping northern Ethiopian communities reduce childhood mortality: population-based intervention trial Bull World Health Organ 2005 83 1 27 33 15682246 PMC2623459

[B37] Harkins T Drasbek C Arroyo J McQuestion M The health benefits of social mobilization: experiences with community-based Integrated Management of Childhood Illness in Chao, Peru and San Luis, Honduras Promot Educ 2008 15 2 15 20 10.1177/1025382308090340 18556732

[B38] Allport G Murchinson C Attitudes A Handbook of Social Psychology 1935 Worcester, Mass.: Clark University Press 798 844

[B39] Huicho L Dávila M Gonzales F Drasbek C Bryce J Victora CG Implementation of the Integrated Management of Childhood Illness strategy in Peru and its association with health indicators: an ecological analysis Health Policy and Planning 2005 20 S1 i32 i41 10.1093/heapol/czi052 16306067

