# Supplementary material for: Health education for microcredit clients in Peru: a randomized controlled trial
Source: BMC Public Health. 2011 Jan 24;11:51. doi: 10.1186/1471-2458-11-51 (PMC3037866; doi:10.1186/1471-2458-11-51)
Supplement: Additional file 1 — CONSORT Flow Diagram. As described in the Methods section, we have included the standard CONSORT checklist and flow diagram to provide further details of the implementation of this study and the interpretation of its results. [file 1471-2458-11-51-S1.DOC]

**CONSORT 2010 Flow Diagram**

**Allocation**

**Analysis**

**Follow-Up**

**Enrollment**

Assessed for eligibility (n= 2,134 )

Excluded (n= 279 )

♦  Not meeting inclusion criteria (n= 0 )

♦  Declined to participate (n= 79 )

♦  Other reasons (n= 200 )

Analysed (n= 757 )
♦ Excluded from analysis (give reasons) (n= 0)

Lost to follow-up (give reasons) (n= 163, due to refusal or absence at time of follow-up survey)

Discontinued intervention (give reasons) (n= )

Allocated to treatment (n= 920 )

♦ Received allocated treatment (n= 920 )

♦ Did not receive allocated treatment (give reasons) (n= )

Lost to follow-up (give reasons) (n= 191, due to refusal or absence at time of follow-up survey )

Discontinued intervention (give reasons) (n= )

Allocated to control (n= 935 )

♦ Received control (n= 935 )

♦ Did not receive control (give reasons) (n= )

Analysed (n= 744 )
♦ Excluded from analysis (give reasons) (n= 0)

Randomized (n= 1,855 )
